# Probiotics and quality of life improvement in cancer therapy

**DOI:** 10.6026/973206300213110

**Published:** 2025-09-30

**Authors:** Himani Muniyal Jheetay, Nikhil Menia, Kishore Chandra Thakur, Seema Gupta

**Affiliations:** 1 Department of Biochemistry, GEIMS, Dehradun, Uttrakhand, India; 2 Department of Pharmacology, AIIMS, Vijaypur, Jammu and Kashmir, India; 3 Department of Anatomy, Graphic Era Institute of Medical Sciences, Dehradun, Uttrakhand, India; 4 Department of Pharmacology GMC Jammu and Kashmir, India

**Keywords:** *Lactobacillus* probiotics, cancer therapy, quality of life

## Abstract

Gastrointestinal toxicity and mental fatigue are common with cancer therapy by reducing patient quality of life. In this observational study patients taking *Lactobacillus* probiotic had better role function (6.52 vs 6.20, p=0.016), emotional balance (11.96 vs 11.58, p=0.020) and cognitive clarity (6.42 vs 6.20, p=0.037) after 6 weeks. Diarrhoea severity was lower (0.78 vs 1.26, p<0.001) and fewer needed rescue loperamide (24% vs 44%, p=0.034). Thus, we show probiotics help patients adapt better to treatment stress and protect gut-brain axis during chemotherapy and radiotherapy.

## Background:

Gastrointestinal toxicities like diarrhoea and mucositis are common during chemoradiotherapy and often reduce treatment adherence, delay cycles and worsen overall patient outcomes [1]. As supportive care options are limited, probiotics are now explored as adjuncts to improve tolerance and outcomes [2]. Probiotic benefits are variable depending on cancer type, host immune response and treatment protocols [2, 3]. Some studies show clear symptom relief while others report modest or no changes. For instance a reduction in severity of oral mucositis was noted in radiotherapy patients given specific probiotic strains [1]. In colorectal cancer, probiotics significantly lowered diarrhoea scores and improved gut barrier integrity during chemoradiotherapy [4]. Head and neck cancer patients showed clinical relief from mucosal side effects using *Lactobacillus* brevis lozenges, suggesting probiotic effects may be site-specific [4]. Another study reported that cancer patients on immunotherapy experienced improved overall quality of life with probiotic intake, indicating benefits beyond gut symptoms [5, 6]. Immunomodulatory effects of probiotics have been discussed as a key mechanism especially in reducing infections and inflammation in cancer patients on cytotoxic therapy [7]. Mechanistic insights further suggest that gut microbiota alterations can influence metabolism of anticancer drugs, affecting both efficacy and toxicity [8]. Despite positive signals, differences in patient profiles, cancer sites, probiotic strains and outcome measures limit generalisability [9]. More studies are needed to confirm actual clinical benefit of probiotics in cancer supportive care [7, 9]. With this background, the present study was conducted to evaluate the effect of *Lactobacillus*-based probiotics on functional quality of life domains in cancer patients undergoing chemotherapy and/or radiotherapy. Therefore, it is of interest to assess changes in physical, role, emotional, cognitive and social functioning using EORTC QLQ-C30 over 6 weeks and compare these domains between probiotic and placebo groups. Additionally, it explored whether probiotics contribute to maintaining daily activity, emotional well-being and social participation during cancer therapy.

## Materials and Methods:

This was a prospective observational study done in Department of Pharmacology and Therapeutics in collaboration with Radiotherapy and Oncology at Government Medical College Jammu. Ethics approval was taken from Institutional Ethics Committee (No. IEC/Pharma/Thesis/Research/2022/970). Written informed consent was obtained from all patients after explaining study in local language. Patients aged 18 to 75 years, diagnosed with gastrointestinal, gynecological or hepatobiliary cancers and starting first course of chemotherapy or radiotherapy were included. Those with previous chemotherapy, radiotherapy, probiotic allergy, active diarrhoea, IBD or severe comorbidities were excluded. Patients were observed under two groups. Group A received standard anticancer therapy with placebo capsules. Group B received same cancer therapy with *Lactobacillus* probiotic 60 million units twice daily for 6 weeks. Both groups were followed clinically and functionally during treatment. Functional quality of life was assessed at start and after 6 weeks using EORTC QLQ-C30 questionnaire. Domains included physical, role, emotional, cognitive and social functioning. Diarrhoea was recorded daily by patients and graded using NCI-CTCAE v5.0. Need for rescue loperamide was also noted. All data were analysed using SPSS version 20. Intergroup comparison was done using unpaired t-test. P-value <0.05 was considered significant.

## Results:

In this study, functional quality of life (QoL) was assessed using EORTC QLQ-C30 in cancer patients receiving chemotherapy or radiotherapy with or without probiotic support. In the placebo group (Group A) only minor changes were seen. Physical function improved from 13.58 ± 2.1 to 14.90 ± 1.9. Role function increased from 5.80 ± 1.3 to 6.20 ± 1.2. Emotional function went up from 10.76 ± 1.6 to 11.58 ± 1.4. Cognitive function changed from 5.94 ± 1.0 to 6.20 ± 0.9. Social function rose from 5.14 ± 0.9 to 5.38 ± 0.8 (Table 1 - see PDF). These gains were small and expected with regular treatment. In the probiotic group (Group B) changes were more marked. Physical function improved from 13.80 ± 2.0 to 15.16 ± 1.6. Role function rose from 6.02 ± 1.2 to 6.52 ± 1.1. Emotional function increased from 10.88 ± 1.7 to 11.96 ± 1.3. Cognitive function improved from 6.06 ± 1.0 to 6.42 ± 0.9. Social function rose from 5.26 ± 0.8 to 5.62 ± 0.7 (Table 2 - see PDF). These improvements were meaningful and suggested better adaptation to treatment. At six week the intergroup comparison showed significant benefit in the probiotic group for role (6.52 vs 6.20, p = 0.016), emotional (11.96 vs 11.58, p = 0.020), cognitive (6.42 vs 6.20, p = 0.037) and social function (5.62 vs 5.38, p = 0.018). Physical function was slightly better in the probiotic group but not statistically significant (15.16 vs 14.90, p = 0.305) (Table 3 - see PDF). Overall the probiotic use was linked with better quality of life in key functional domains during cancer therapy.

## Discussion:

This observational study shows that *Lactobacillus* probiotic use during chemotherapy or radiotherapy improves functional quality of life, mainly in role, emotional and cognitive domains. Over six weeks patients on probiotics had better EORTC QLQ-C30 scores, indicating that probiotics may help reduce symptom load and support mental clarity during cancer therapy. The probiotic group had significantly lower diarrhoea scores (0.78 ± 0.507) than placebo (1.26 ± 0.487, p < 0.001) and fewer patients required rescue medication (24% vs 44%, p = 0.034). These findings agree with Yang *et al.* who reported lower diarrhoea frequency in colorectal cancer patients treated with *Lactobacillus* casei Shirota during chemoradiotherapy [10]. Mego *et al.* also observed fewer grade II/III diarrhoea cases in patients given probiotics, suggesting gut protection [7]. Bartsch *et al.* in their meta-analysis noted that pre-, pro- and synbiotics reduced radiation-induced diarrhoea, with a pooled RR of 0.71 (95% CI: 0.52-0.97), supporting our results 11]. Emotional and cognitive benefits seen here align with findings by Lee *et al.*, where newly diagnosed breast cancer patients with healthier gut microbiota showed reduced salivary cortisol and better emotional control, indicating gut-brain axis involvement [12]. Naeem *et al.* proposed that probiotics downregulate inflammatory cytokines and enhance gut lining, improving mood and reducing fatigue during cancer treatment [13].

Physical and social domains didn't improve much in our cohort. A similar selective benefit was reported by in a work where *Lactobacillus* plantarum 299v improved GI tolerance but showed inconsistent effects across all QoL subdomains due to underlying nutritional variability and disease stage [14]. Bai *et al.* confirmed that baseline microbiome differences influence radiation-related gut toxicities, which explains interindividual variation [15]. Some opposing observations were noted too. Folwarski *et al.* in a placebo-controlled trial using *Lactobacillus* plantarum 299v found improved GI tolerance but no significant QoL improvement, possibly due to nutritional confounders [14]. Thomsen *et al.* in a real-world oncologic cohort during COVID-19 found probiotic benefits on inflammation and gut function but subjective QoL improvements varied and were not statistically robust [16]. Our findings support that *Lactobacillus* probiotics during active cancer therapy help in maintaining role functioning, reducing emotional fatigue and preserving cognitive clarity. Though causality can't be claimed from observational data, consistent benefits reported across multiple studies build a strong case. Probiotics remain a low-cost, safe and promising adjunct in supportive oncology care. Sliżewska *et al.* added mechanistic support by showing that probiotic strains restore intestinal microbiota enhances short-chain fatty acid production and reduce inflammatory markers in cancer patients on therapy [17]. On the other hand in lung cancer patients oral probiotic supplements improved quality of life but benefits were more pronounced in emotional and social well-being than in physical domains, indicating site and population-specific responses [18]. These mixed patterns suggest probiotics act differently across cancers and patient backgrounds.

## Conclusion:

*Lactobacilli* probiotics help improve functional quality of life in cancer patients on chemotherapy or radiotherapy. Role, emotional and cognitive domains showed significant gains over 6 weeks with probiotic use is shown. Diarrhoea severity and rescue drug need were both lower in the probiotic group. Improvements seen suggest gut support may reduce treatment stress and mental fatigue. Probiotics appear safe, low-cost and clinically helpful in supporting cancer therapy especially in clinical settings.
